# Acquisition and Assimilation of Nitrogen as Peptide-Bound and D-Enantiomers of Amino Acids by Wheat

**DOI:** 10.1371/journal.pone.0019220

**Published:** 2011-04-26

**Authors:** Paul W. Hill, Richard S. Quilliam, Thomas H. DeLuca, John Farrar, Mark Farrell, Paula Roberts, Kevin K. Newsham, David W. Hopkins, Richard D. Bardgett, David L. Jones

**Affiliations:** 1 Environment Centre Wales, Bangor University, Bangor, Gwynedd, United Kingdom; 2 Soil and Ecosystem Ecology Laboratory, Lancaster Environment Centre, Lancaster University, Lancaster, United Kingdom; 3 Commonwealth Scientific and Industrial Research Organisation Land and Water, Glen Osmond, South Australia, Australia; 4 Ecosystems Programme, British Antarctic Survey, Natural Environment Research Council, High Cross, Cambridge, United Kingdom; 5 Scottish Crop Research Institute, Invergowrie, Dundee, United Kingdom; 6 School of Biological and Environmental Sciences, University of Stirling, Stirling, United Kingdom; 7 School of Life Sciences, Heriot-Watt University, Riccarton, Edinburgh, United Kingdom; University of Leipzig, Germany

## Abstract

Nitrogen is a key regulator of primary productivity in many terrestrial ecosystems. Historically, only inorganic N (NH_4_^+^ and NO_3_^-^) and L-amino acids have been considered to be important to the N nutrition of terrestrial plants. However, amino acids are also present in soil as small peptides and in D-enantiomeric form. We compared the uptake and assimilation of N as free amino acid and short homopeptide in both L- and D-enantiomeric forms. Sterile roots of wheat (*Triticum aestivum* L.) plants were exposed to solutions containing either ^14^C-labelled L-alanine, D-alanine, L-trialanine or D-trialanine at a concentration likely to be found in soil solution (10 µM). Over 5 h, plants took up L-alanine, D-alanine and L-trialanine at rates of 0.9±0.3, 0.3±0.06 and 0.3±0.04 µmol g^−1^ root DW h^−1^, respectively. The rate of N uptake as L-trialanine was the same as that as L-alanine. Plants lost *ca.*60% of amino acid C taken up in respiration, regardless of the enantiomeric form, but more (*ca.*80%) of the L-trialanine C than amino acid C was respired. When supplied in solutions of mixed N form, N uptake as D-alanine was *ca.*5-fold faster than as NO_3_^-^, but slower than as L-alanine, L-trialanine and NH_4_^+^. Plants showed a limited capacity to take up D-trialanine (0.04±0.03 µmol g^−1^ root DW h^−1^), but did not appear to be able to metabolise it. We conclude that wheat is able to utilise L-peptide and D-amino acid N at rates comparable to those of N forms of acknowledged importance, namely L-amino acids and inorganic N. This is true even when solutes are supplied at realistic soil concentrations and when other forms of N are available. We suggest that it may be necessary to reconsider which forms of soil N are important in the terrestrial N cycle.

## Introduction

Nitrogen is a key factor in the control of carbon fixation by photosynthetic primary producers [1], [2]. Historically, higher plants were thought to be dependent on inorganic N (NH_4_^+^ and NO_3_^-^) for all of their N requirements. However, in the absence of human inputs of synthetic inorganic N, most N enters soil as protein, and this remains the dominant form of soil organic N [3]–[5]. Consequently, plant productivity in N-limited ecosystems was thought to be controlled by the rate of microbial mineralization of organic N to inorganic N. In the 1990s our understanding of the regulation of plant productivity was revolutionised by the demonstration of a “short-circuit” in the N cycle. Plants were shown to take up L-enantiomers of amino acids [6], [7] with productivity being limited by the rate of microbial protein/peptide cleavage to amino acids. The importance of L-amino acids to the N cycle has subsequently received a great deal of interest [7]. However, soil soluble N is as abundant as small peptides (<1 kDa MW) as it is as free amino acids (Table 1) [8], [9]. Despite the identification of peptide transporters in various plant tissues including roots, there has been surprisingly little consideration of the nutritional and ecological significance of plants competing for N at an earlier stage of protein cleavage than free amino acids [9]–[13].

**Table 1:** Concentrations of inorganic, amino acid and peptide N in the soil solution of a UK agricultural soil^a^.

	N concentration (µmol N l^−1^)
Total dissolved N	844±30
Total dissolved N <1 kDa	746±46
Peptidic-N <1 kDa	31±2
Free amino acid N	4±0.9
NH_4_^+^	16±4
NO_3_^-^	655±38

a Values are mean ± SEM; *n* = 4.

Short peptides of D-amino acids are essential components of bacterial peptidoglycan and some D-amino acids exist in soil organic matter at 10 to 20% of the concentration of L-enantiomers [14], [15]. There is some existing evidence that plants are able to metabolise D-amino acids, and D-amino acids and amino acid racemases have been reported in plants [16]–[20]. Nevertheless, some reports of phytotoxic effects of certain D-amino acids (e.g. D-serine), when supplied at high concentrations relative to those in soil, have resulted in D-amino acids being discounted as important plant N resources [7], [16], [21], [22]. D-peptides have been reported in plant tissues [19], [20], but very little information exists on the capacity of plants to take up and assimilate them through their roots [23].

We conducted a straightforward test of the effect of polymeric and enantiomeric form on the uptake and assimilation of amino acid N supplied to a higher plant in the absence of mycorrhizal symbionts. We directly compared D- and L-forms of the same amino acid, and the D- and L-forms of their corresponding tripeptides, to test the hypothesis that non-symbiotic higher plants are able to take up and assimilate amino acids and small peptides supplied at the low concentrations likely to be present in soil solution, irrespective of entantiomeric form. We further compared rates of uptake of these organic forms of N with those of inorganic forms of N. As a conservative test of organic N use, we chose an agricultural plant, wheat, which has been bred to grow with high inputs of synthetic inorganic N. As the amino acid monomer, we chose alanine which is common in all kingdoms of organisms as an individual amino acid and short homopeptides, and in soil as both L- and D-enantiomers [14], [15], [24].

## Results and Discussion

**Figure 1:**
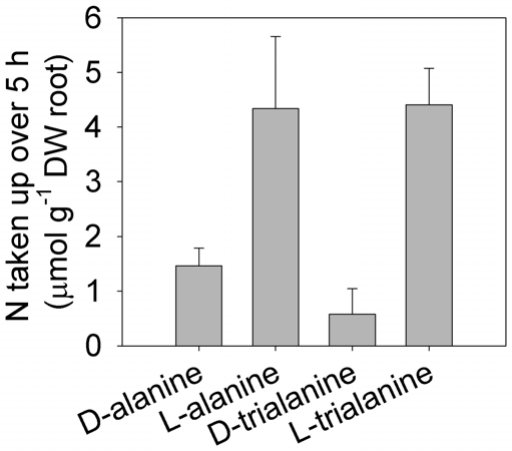
Uptake of peptide or amino acid N by sterile roots of wheat. Uptake determined over 5 h from the depletion of ^14^C from 10 µM solutions of single N forms. Values are mean ± SEM; *n* = 3.

Over 5 h, sterile roots of wheat took up ^14^C-labelled L-alanine, D-alanine and L-trialanine at rates of 0.9±0.3, 0.3±0.06, and 0.3±0.04 µmol g^−1^ DW root h^−1^, respectively (mean ± SEM; *n* = 3) from a 10 µM solution reflecting realistic soil solution concentrations. There was no difference in the rate of N uptake as L-trialanine and that as L-alanine (Fig. 1). Plants took up 80 to 90% less (*P*<0.05) D-peptide than other forms of organic N. D-trialanine was taken up at a rate of only 0.04±0.03 µmol g^−1^ DW root h^−1^. Recovery of plant ^14^C by combustion revealed that ^14^C was translocated and 66±5, 58±5 and 83±4% (L-alanine, D-alanine and L-trialanine, respectively) of substrate ^14^C removed from solution was lost in respiration (not recovered in plant tissues). The ^14^C recovered in plants exposed to D-trialanine was the same as that removed from solution and a much higher (*P*<0.001) proportion of D-trialanine ^14^C was recovered in the shoot than in the root in comparison to other substrates. Although possibly not accurately representing the partitioning of N, the ratio of ^14^C recovered in the root to ^14^C recovered in the shoot was 6.0±2, 4.6±0.4, 5.7±1.6 and 0.5±0.01 for D-alanine, L-alanine, L-trialanine and D-trialanine, respectively. This indicates that plants took up and assimilated L- and D-amino acids and L-peptide, but were unable to assimilate even the small quantity of D-peptide taken up. The *ca.*20-fold difference between L-alanine uptake and the uptake of D-trialanine is consistent with the previously reported 20-fold difference found in uptake of amino acids between control plants and those treated with protonophores e.g. CCCP [25]. Consequently, we suggest that uptake of D-trialanine was by passive uptake alone.

**Figure 2:**
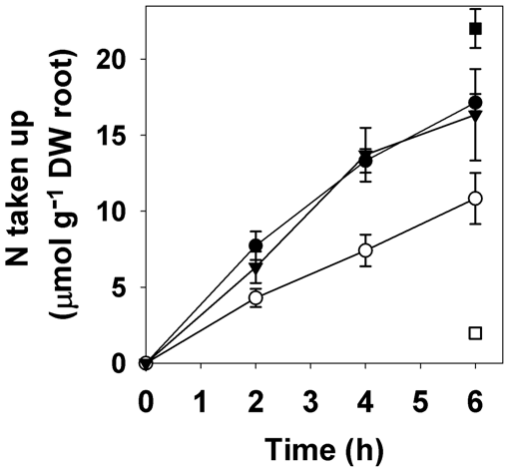
Uptake of N by sterile roots of wheat from a mixed N form solution. Uptake determined by solution ^14^C depletion (organic N) or ^15^N recovery in plants (inorganic N). L-alanine •, D-alanine ○, L-trialanine ▾, NO_3_^-^ □, NH_4_^+^ ▪. Values are mean ± SEM; *n* = 3.

When other forms of N were available to plants in an equimolar solution containing five forms of N (L-alanine, D-alanine, L-trialanine, KNO_3_ and NH_4_Cl), N was taken up as the D-amino acid monomer at a five-fold higher (*P* = 0.004; Fig. 2) rate than NO_3_^-^. Uptake of N as D-alanine was, however, 37% slower (*P*≤0.04) than as L-alanine, which was taken up at the same rate as L-trialanine N and NH_4_^+^. Rates of metabolism of L-peptide and L- and D-amino acids, as determined from losses of ^14^C in respiration, were the same when acquired from the mixed solution as when N forms were supplied individually. In both cases, the proportion of the ^14^C taken up which was respired by plants was greatest (*P*≤0.03) when supplied as L-trialanine. This *ca.*25% increase in post-uptake metabolism between peptides and their amino acid monomers strongly suggests that there was no extracellular cleavage of peptides prior to uptake.

Organic N uptake has been identified as important in natural habitats [6], [7], [9], [26], [27]. However, our results show that even plants such as wheat, bred to grow with high inorganic N additions, can take up and assimilate peptide N at a rate comparable to those of N forms of known importance for plant nutrition, namely L-amino acid and NH_4_^+^, and greatly exceeding that of NO_3_^-^. This is true even when peptides are supplied at low soil concentrations and when other forms of N are available to the plant. The concentration of solutes in soil is maintained by the balance between their input or production, and their consumption by soil microorganisms and plants. Consequently, successful root uptake and assimilation of peptides when supplied at the low concentrations maintained in soil, strongly suggests that plants are capable of competing with soil microorganisms for N at an early stage of protein decomposition. Thus, the rate-limiting step in N-limited plant productivity may be the rate of protein cleavage to short peptides rather than the rate of protein/peptide cleavage to free amino acids or the rate of microbial mineralisation of amino acids to inorganic N. There is some evidence that plants may be able to take up intact protein through their roots, but quantities appear to be very low [28]. Consequently, uptake of peptides very likely represents the uppermost level of plant competition with soil microbes for N resources.

Plants are apparently unable to utilise D-peptide N, assuming D-trialanine and wheat are representative. However, our data show that they are clearly able to take up and assimilate D-alanine when supplied at soil solution concentrations and do so in preference to NO_3_^-^. As D-amino acids, such as D-alanine, are common in bacteria and in soil, we suggest that they may be more important as a source of N to plants than has previously been recognised. We further suggest that the often relatively high concentrations of NO_3_^-^ in soil solution [29] (Table 1) may not reflect its importance to plants as a large pool of available N, but rather the preference of plants for other forms of N, which leads to slower depletion of the soil NO_3_^-^ pool.

These findings indicate that plants can acquire and metabolise N in forms that are not currently considered to be of importance for plant nutrition, and at an earlier stage in the N cycle than previously thought. Further, such early uptake of more complex soil N by plants must necessarily affect the availability of substrate for downstream microbial N transformations and the flux of N through soil pools. There are many possible variations in peptide composition, and much further work is necessary to fully elucidate the relative importance of the various forms of soil N available to plants. Nevertheless, we suggest that it may be necessary to reconsider current assumptions concerning the fundamental pattern of N flow in the plant-microbe-soil continuum.

## Materials and Methods

### Soil solution characterisation

Agricultural soil was collected from a depth of 0–10 cm in four locations at Bangor University's Henfaes Research Station (53° 14′N, 4° 01′W). Background soil characteristics are given in [30]. Soil solution was extracted by centrifugal drainage [31], sterilised by filtration to 0.2 µm and passed through a 1 kDa ultrafiltration membrane (Millipore, Billerica, MA, USA). Amino acid N was measured fluorometrically according to [32] before and after hydrolysis in 6 M HCl at 105°C for 16 h under N_2_. Total dissolved N was measured in a TOC-V-TN analyzer (Shimadzu, Kyoto, Japan). Nitrate and ammonium were measured colorimetrically according to [33] and [34], respectively.

### Uptake from solutions of single N forms

Seeds of wheat (*Triticum aestivum* L. cv. Claire) were surface sterilised in 10% NaClO followed by 80% ethanol, and grown in Phytatrays (Sigma Aldrich, Gillingham, UK) on 10% Murashige and Skoog agar in natural light. At the third leaf stage, roots of single plants (*n* = 3) were placed in 4 ml of sterile (0.2 µm-filtered) solutions of either 10 µM, *ca.*1.5 kBq U^-14^C-labelled, L-alanine (C_3_H_7_NO_2_), D-alanine, L-trialanine (C_9_H_17_N_3_O_4_), or D-trialanine (unlabelled from Bachem, Bubendorf, Switzerland; labelled from American Radiolabeled Chemicals, St Louis, MA, USA). All operations were carried out aseptically in a laminar flow cabinet at *ca.*25°C and a light intensity of 170 µmol photons m^-2^ s^-1^ PAR. After 5 h, plants were washed in deionised water for *ca.*1.5 min and the remaining ^14^C activity of solutions was measured by liquid scintillation counting in a Wallac 1404 scintillation counter (Perkin-Elmer, Boston, MA, USA). Plants were dried at 80°C, before combustion in an OX400 biological oxidizer (RJ Harvey, Hillsdale, NJ, USA). Liberated ^14^CO_2_ was captured in Oxosol scintillant (National Diagnostics, Atlanta, GA, USA) and measured by liquid scintillation counting.

### Uptake from solutions of mixed N-forms

Plant roots were placed in 4.5 ml of a mixed N form solution of L-alanine, D-alanine, L-trialanine, NH_4_Cl and KNO_3_. Each of 3 replicates had one N form labelled with either *ca.*4 kBq ^14^C (peptide and amino acids) or 98 atom % ^15^N (NH_4_^+^ and NO_3_^-^; Sigma Aldrich, Gillingham, UK). In this case, substrates were all supplied at a concentration of 50 µM to ensure that sufficient ^15^N for accurate measurement could be recovered in plants. Aliquots of 50 µL were removed after 2, 4 and 6 h and ^14^C activity measured by liquid scintillation counting where appropriate. After 6 h plants were washed for *ca.*2 min in 0.1 M CaCl_2_. The ^14^C activity of washings was measured. Plants were dried and combusted in the biological oxidizer or ground and analyzed for ^15^N in a Eurovector EA-Isoprime IRMS (Eurovector SpA, Milan, Italy) as appropriate. All methods and conditions were as described for uptake from solutions of single N-form, except where stated.

### Statistical analysis

All statistical analysis by one-way ANOVA with LSD post-hoc test (SPSS v14, SPSS Inc, Chicago, USA).
